# Neurodegenerative Biomarkers in Multiple Sclerosis: At the Interface Between Research and Clinical Practice

**DOI:** 10.3390/diagnostics15091178

**Published:** 2025-05-06

**Authors:** Alin Ciubotaru, Mădălina Irina Smihor, Cristina Grosu, Daniel Alexa, Roxana Covali, Robert-Constantin Anicăi, Ioana Păvăleanu, Andrei Ionuț Cucu, Amelian Mădălin Bobu, Cristina Mihaela Ghiciuc, Emilian Bogdan Ignat

**Affiliations:** 1 Department of Neurology, “Grigore T. Popa” University of Medicine and Pharmacy, 700115 Iasi, Romania; alinciubotaru94@yahoo.com (A.C.); cristina.grosu@umfiasi.ro (C.G.); alexadaniel2004@yahoo.com (D.A.); emilian.ignat@umfiasi.ro (E.B.I.); 2 Grigore T. Popa, University of Medicine and Pharmacy, 700115 Iasi, Romania; mg-rom-31105@students.umfiasi.ro (M.I.S.); anicairobert98@gmail.com (R.-C.A.); 3 Department of Radiology, Biomedical Engineering Faculty, “Grigore T. Popa” University of Medicine and Pharmacy, 700115 Iasi, Romania; 4 Mother and Child Department, “Grigore T. Popa” University of Medicine and Pharmacy, 700115 Iasi, Romania; ioana-m-pavaleanu@umfiasi.ro; 5 Faculty of Medicine and Biological Sciences, University Stefan cel Mare of Suceava, 720229 Suceava, Romania; andrei.cucu@usm.ro; 6 Department of Cardiology, Sf. Spiridon Hospital, 700111 Iași, Romania; amelian.bobu@gmail.com; 7 Department of Morpho-Functional Sciences II—Pharmacology and Clinical Pharmacology, “Grigore T. Popa” University of Medicine and Pharmacy, 700115 Iasi, Romania; cristina.ghiciuc@umfiasi.ro; 8 “Saint Mary” Emergency Children Hospital, 700887 Iasi, Romania

**Keywords:** multiple sclerosis, neurofilaments, neurodegeneration, kynurenines

## Abstract

Multiple sclerosis (MS) is a chronic autoimmune disorder characterized by inflammation, demyelination, and neurodegeneration within the central nervous system (CNS). While the inflammatory components of MS have been extensively studied, the progressive neurodegenerative aspect remains a critical factor contributing to long-term disability. Therefore, the identification and validation of biomarkers associated with neurodegenerative processes are essential for improved diagnosis, prognosis, and treatment monitoring. This review explores cerebrospinal fluid (CSF) and blood-based biomarkers, including neurofilaments, lipid markers, kynurenines, and other molecular indicators that provide insights into neurodegeneration in MS.

## 1. Introduction

Multiple sclerosis (MS) is a multifaceted disorder that affects both white and gray matter structures in the central nervous system (CNS). Traditionally regarded as an inflammatory demyelinating disease, recent studies have underscored the importance of neurodegeneration in MS pathology. In contrast to relapsing–remitting MS (RRMS), where inflammatory processes dominate, the progressive forms of MS show the predominance of neurodegeneration processes. The development of biomarkers capable of distinguishing between inflammatory and neurodegenerative changes during the evolution of the disease are crucial for the optimization of therapeutic strategies.

The number of people affected worldwide is estimated at around 2.8 million with the highest prevalence observed in Europe. Multiple sclerosis is the most common chronic neurological disease among young adults, predominantly affecting women, with a mean age of onset of 30 years. The economic and social costs associated with MS, including loss of productivity and long-term healthcare needs, highlight the importance of early detection and intervention. A comprehensive understanding of the neurodegenerative components of MS is crucial for the development of targeted therapeutic interventions aimed at decelerating that can slow disease progression. Moreover, the heterogeneity of MS presents challenges in predicting individual disease courses, reinforcing the need for comprehensive biomarker profiling to personalize treatment strategies effectively.

In recent years, a considerable body of literature has focused on the identification and validation of neurodegenerative biomarkers in multiple sclerosis (MS). While numerous reviews have discussed the potential utility of various biomarkers, few have systematically synthesized quantitative data regarding their diagnostic performance. The present review aims to address this gap by providing a comprehensive, cohort-specific synthesis of sensitivity and specificity values for key emerging biomarkers, including neurofilament light chain (NfL), glial fibrillary acidic protein (GFAP), total tau (t-tau), and others. By integrating detailed cohort characteristics and critically highlighting areas where data remain insufficient, this work not only offers a practical guide for clinicians but also delineates directions for future research. Furthermore, by contextualizing these findings within clinical practice, the present article seeks to enhance the interpretability and applicability of biomarker data.

### 1.1. Risk Factors of Progression to Secondary Progressive Multiple Sclerosis

There are multiple clinical and demographic risk factors for the progression from relapse-remitting multiple sclerosis (RRMS) to secondary progressive multiple sclerosis (SPMS). Older age at disease onset is correlated with an increased risk of conversion. Furthermore, higher Expanded Disability Status Scale (EDSS) scores at onset and the presence of spinal cord lesions and cerebellar dysfunction at diagnosis are correlated with an increased risk of disease progression. Smoking was also shown to be a risk factor for transitioning to SPMS [1].

From a neurobiological perspective, neuronal exhaustion plays a role in the disease’s advancement, highlighted by higher neurofilament light chain levels and accelerated gray matter atrophy. Furthermore, patients who experience high relapse rates in the early stages of the disease are at greater risk of progressing to SPMS [2].

### 1.2. Objective of This Review

The objective of this review is to summarize and collect actual data about the present and possible future neurodegenerative biomarkers for either diagnostic or progression monitoring used in MS to offer a better understanding of the subject. We will evaluate the neurodegenerative biomarkers found in CSF, blood, and their correlation with the clinical evaluation of diagnosis and progression measured by disability. It is important to note that, in addition to the inflammatory biomarkers, there are multiple promising biomarkers displaying neurodegeneration in multiple sclerosis (MS). However, these will not be addressed in this review.

The objective of this study is to elucidate the function of each neurodegenerative biomarker that has been examined and its potential application in conjunction with MRI and clinical assessments of disease progression as a decision-support tool for managing patients with MS [3].

Using multiple biomarkers may prove to be useful in developing a more comprehensive panel that addresses the limitations of using a single biomarker. Consequently, more research with recent technological and statistical approaches is needed to identify novel and effective diagnostic and prognostic biomarker tools in MS [4].

### 1.3. Materials and Methods

We conducted a comprehensive literature review focused on neurodegenerative biomarkers in MS. The search strategy included querying PubMed and relevant databases for clinical trials, cohort studies, and meta-analyses published in the last two decades that evaluated biomarkers of neuronal or axonal injury in MS. Keywords used in the search encompassed terms such as “multiple sclerosis biomarkers”, “neurodegeneration”, “axonal damage markers”, and specific biomarker names (e.g., “neurofilament”, “tau protein”, “NfL”, “GFAP”, “YKL-40”, “chitinase”, “NSE”, “14-3-3”, “kynurenine”, “myelin basic protein”, “extracellular vesicles”, “metabolomics”, and “lipid biomarkers”). Both cerebrospinal fluid and blood-based biomarkers were included. We also included relevant systematic reviews and meta-analyses to ensure comprehensive coverage of the topic. The inclusion criteria for studies were as follows: (1) studies involving patients with MS (any subtype) that measured one or more of the candidate neurodegenerative biomarkers; (2) reporting on sensitivity, specificity, or clinical correlations (e.g., with disease activity, progression, or outcomes); and (3) published in peer-reviewed journals. Data were extracted on study design, sample size, MS phenotype, the biomarker levels in MS vs. controls, and any measures of diagnostic performance (sensitivity, specificity) or prognostic significance. Given the heterogeneity of methods, a qualitative synthesis was performed rather than a formal meta-analysis (except where meta-analytic data were available from the literature). The findings for each biomarker are summarized in the Results Section.

### 1.4. Glossary of Terms

- Diagnostic biomarker: A biomarker used to indicate or confirm the presence of disease (MS), aiding in the diagnosis (e.g., distinguishing MS from other conditions at disease onset).
- Prognostic biomarker: A biomarker that predicts the future disease course or outcome (for example, risk of relapses, rate of disability progression, or conversion to SPMS).
- Monitoring biomarker: A biomarker that reflects current disease activity or treatment response, used for ongoing tracking of MS and to inform adjustments in therapy.

## 2. Key Biomarkers in MS and Their Clinical Significance

There are a variety of biomarkers currently under investigation for their potential to track neurodegeneration, predict disease progression, and assess treatment response in MS. We will discuss each biomarker in detail, outlining its role and clinical relevance. According to the World Health Organization (WHO), a biomarker is defined as “any substance, structure, or process that can be measured in the body or its products and influence or predict the incidence of outcome or disease”. The characteristics of a good biomarker need to fit certain criteria: it must be easily reproducible and inexpensive; it must have good specificity and sensitivity; it must have the ability to produce reliable results; and it must be obtained through minimal/not-invasive ways [5].

**Current Biomarkers:**

### 2.1. Biomarkers of Axonal Damage

#### 2.1.1. Neurofilament Light Chain (NfL)

Neurofilament light chain (NfL) is a well-established biomarker of axonal damage and neurodegeneration. NfL is released into the cerebrospinal fluid (CSF) and blood following neuronal injury, making it a reliable indicator of disease activity. Elevated NfL levels correlate with brain atrophy, disability progression, and response to treatment [6]. As a result, NfL is increasingly used to monitor the effectiveness of disease-modifying therapies (DMTs) in MS patients [7]. High baseline sNfL levels have been linked to a higher risk of future relapses and new MRI lesions [5]. In other words, an MS patient with very elevated sNfL levels at onset is likely experiencing subclinical disease activity, which may signal suboptimal disease control if not addressed. One of the limitations of the serum NfL is that it is specific for pathology (i.e., neuronal damage) but not for a specific disease. Elevated levels have been reported in acute conditions such as traumatic brain injury or stroke and in chronic neurodegenerative diseases such as Alzheimer’s disease and frontotemporal dementia. Therefore, individual values can only be interpreted within the clinical context [7]. NfL measurements may also serve as a complementary tool to expedite diagnosis and prognostication. For example, an elevated NfL level after a first demyelinating event (RIS or CIS) could foreshadow a faster conversion to clinically definite MS, potentially justifying earlier intervention [5]. Serum NfL (sNfL) has certain advantages over traditional measures of MS disease progression such as MRI because it is relatively noninvasive, inexpensive, and can be repeated frequently to monitor activity and treatment efficacy. sNfL levels can be monitored regularly in patients with MS to determine change from baseline and predict subclinical disease activity, relapse risk, and the development of gadolinium-enhancing (Gd+) lesions [3]. However, inter-assay variability remains an issue for sNfL. For example, a comparison of SiMoA vs. high-sensitivity ELISA for sNfL found only a moderate correlation between platforms and significant biases in measured levels [8]. This highlights the need to standardize NfL assay methods and reference ranges across laboratories for consistent clinical implementation.

#### 2.1.2. Tau Protein

Tau protein, which is commonly associated with Alzheimer’s disease, is also emerging as a potential biomarker in MS. In MS, studies of CSF total tau (t-tau) have yielded mixed results. Some patients with early or active MS exhibit mildly increased CSF tau compared to controls, reflecting axonal damage, but levels are generally much lower than those seen in primary neurodegenerative dementias [9]. The sensitivity of t-tau for MS is low to moderate—many MS patients have normal CSF tau, especially outside of acute relapses [10]. Phosphorylated tau (p-tau), a hallmark biomarker in Alzheimer’s, does not typically show a specific elevation in MS except in cases where an MS patient has coexisting Alzheimer pathology. One study reported that a subset of progressive MS patients with severe cognitive impairment had abnormal CSF p-tau and Aβ profiles, suggesting a concurrent neurodegenerative process [10]. Thus, tau is more useful in ruling out other diagnoses than as a primary MS marker [11,12]. Hyperphosphorylated tau species have also been detected in MS and correlated with cognitive impairment and disease severity [13].

#### 2.1.3. Amyloid—Precursor Protein (APP)

APP is produced by astrocyte cells during demyelination and can be located in reactive glial cells during de- and remyelination. Higher APP levels are present in MS patients and correlate with CNS lesion development [4]. In MS, APP is not measured in biofluids as a soluble biomarker but examined in tissue as an indicator of acute axonal damage. Histopathological studies have shown that transected axons in acute MS lesions often exhibit accumulations of APP as a reaction to injury [14]. Gehrmann et al. first reported APP expression within MS plaques, signifying ongoing axonal transection. The presence of APP immunoreactive axonal spheroids in biopsy or autopsy specimens of MS confirms that active demyelination is accompanied by neurodegeneration [15]. Thus, while APP servers as a qualitative tissue biomarker of axonal damage in MS lesions, it is not used diagnostically in live patients. Its significance lies in the pathological evidence of neurodegeneration in MS.

#### 2.1.4. Tubulin Beta (TUB β)

TUB β are heterodimeric proteins that make up microtubules. Neuron regeneration is associated with increased production of the class ll tubulin isotype and increased in MS patients compared to patients with other neurological diseases [4]. However, tubulin beta is not a well-established MS biomarker at present. Some proteomic studies have identified tubulin peptides in CSF when comparing MS patients to controls, suggesting that it is part of a panel of injury-related proteins [14]. The sensitivity or specificity of tubulin beta for MS has not been clearly reported in the literature, likely due to insufficient focused studies. As a structural protein, tubulin is abundant in the CNS, but distinguishing whether its presence in CSF is MS-specific or due to non-specific neural damage is challenging. Therefore, tubulin beta remains an exploratory biomarker. It may be considered in future panel assays or targeted analyses of neuronal exosomes, but, currently, it has no confirmed clinical utility in MS.

### 2.2. Biomarkers of Neuronal Damage

#### 2.2.1. 14-3-3 Protein

14-3-3 protein, which is present in neurons, can be measured in the CSF of both patients with MS and those with Creutzfeldt–Jakob disease. However, the role of 14-3-3 protein in MS is inconsistent. Studies have found that 14-3-3 protein in the CSF is associated with more severe disability, more extensive involvement of the spinal cord, and quicker progression to MS or disease progression. The early accumulation of 14-3-3 protein in the CSF may be related with decreased rates of recovery [4]. 14-3-3 is typically not detectable in most MS patients, as the sensitivity of 14-3-3 overall is low (only a minority of MS cases are positive), but, when present, it has a high specificity for significant CNS tissue damage (since 14-3-3 rarely appears, except in conditions causing substantial neuronal death). Therefore, a positive 14-3-3 in an MS patient could serve as a red flag for an atypically aggressive course [4]. However, due to its low prevalence, 14-3-3 is not used as a routine biomarker in MS.

#### 2.2.2. Neuron Specific Enolase

NSE is a glycolytic enzyme found in neurons, and elevated levels in CSF or blood indicate neuronal injury. In MS, NSE has been studied as a potential marker of neuroaxonal damage. Research findings have shown that NSE levels are modestly higher in MS patients compared to healthy controls, particularly during relapses or in progressive phases, though the differences are not as pronounced as with NfL [14,16]. The sensitivity of NSE for detecting MS activity is moderate; one large study found that serum NSE was not a strong predictor of disability progression in relapsing MS, but, in primary progressive MS patients, there was a moderate correlation between higher NSE levels and worse disability scores [16]. Specificity is also moderate, since NSE can be elevated in various neurological conditions (e.g., stroke, head injury). Overall, NSE alone appears to be a weaker biomarker in MS than NfL. Its levels tend to increase with age and other factors, which confounds its utility [16]. While elevated NSE in CSF may confirm the occurrence of neuronal damage in MS (especially if other markers are unavailable), its clinical use is limited due to lower sensitivity and specificity. Some authors have suggested NSE could be part of a panel of biomarkers, but, on its own, it has not proven highly informative [12]. Concentrations of neuron specific enolase (NSE), an enzyme found in neurons and axons that can be used to estimate neuronal density, have been found to be increased in both the CSF and serum of patients suffering from trauma, hypoxic brain injury, or cerebral bleeding. However, its role in MS is rather inconsistent since several studies showed no difference between MS patients and the control [4].

### 2.3. Biomarkers of Glial Dysfunction

#### 2.3.1. Glial Fibrillary Acidic Protein (GFAP)

GFAP is an astrocytic marker that reflects astrocyte activation and gliosis in MS. High GFAP levels are associated with neuroinflammation and progressive MS phenotypes. This biomarker is particularly useful in distinguishing MS subtypes. Recent ultrasensitive assays have enabled the measurement of GFAP in serum; studies have found that serum GFAP is higher in progressive MS patients compared to those with relapsing–remitting MS, and higher GFAP associates it with more advanced disease [12,17]. GFAP appears to have high specificity for progressive neurodegeneration in MS—in other words, elevated GFAP is unlikely in purely inflammatory states without significant tissue injury [18]. Higher serum and CSF GFAP levels were found to prognosticate worse outcomes of progression as well as brain atrophy, especially in the gray matter and independent of serum NfL, making it an important biomarker particularly because it is less associated with the acute clinical phase of the disease. Serum GFAP levels tend to rise during relapses in relapsing–remitting MS compared to periods of remission while being able to predict confirmed disability progression even in non-active MS cases [7]. The current limitation is establishing standardized reference ranges. Nonetheless, GFAP’s potential has led to its inclusion in biomarker research panels and its consideration for use alongside NfL in managing progressive MS.

#### 2.3.2. Chitinase-3-like Protein 1 (CHI3L1, Also Known as YKL-40)

CHI3L1, also known as YKL-40, is a marker of glial activation and neuroinflammation. Increased CSF CHI3L1 levels have been linked to a more severe disease course and worse prognosis in MS patients. CHI3L1 has been proposed as a biomarker for distinguishing patients with aggressive MS who may require early intervention with high-efficacy treatments [12,14]. YKL-40 appears to reflect a component of the disease related to inflammation and tissue remodeling by glia. In terms of performance, CSF YKL-40 has moderate sensitivity for MS disease activity (it may not rise during every relapse but tends to be higher in patients with a more aggressive disease course) and moderate specificity (it is also elevated in other neurological conditions involving neuroinflammation, such as CNS infections or neurodegenerative diseases) [14]. However, some studies have identified YKL-40 as an independent predictor of disability progression in MS, even when accounting for NfL levels. This suggests that combining markers of axonal damage (NfL) with markers of glial activation (YKL-40) could improve prognostic accuracy. Overall, YKL-40 is a promising biomarker for the “slow burn” astroglial pathology in MS and may help identify patients at the risk of progression. Ongoing research is working to validate cutoff values and to standardize assays so that YKL-40 might be used clinically alongside NfL in the future.

#### 2.3.3. S100B Protein

S100B is a calcium-binding protein produced predominantly by astrocytes (and to some extent by oligodendrocytes) that serves as a marker of blood–brain barrier dysfunction and glial activation. Increased serum and CSF levels of S100B have been linked to neuroinflammation and neurodegeneration in MS. Consequently, it has the potential to serve as a useful biomarker for monitoring disease activity and treatment response [12]. CSF S100B levels have been found to be slightly elevated compared to controls, particularly during active disease phases. The reported sensitivity of CSF S100B for MS is modest (~65–75%), and specificity is also around 70–80% [7]. These values indicate that S100B alone is not a strong discriminator, but elevated S100B does suggest the presence of CNS damage. Some studies have linked higher S100B to gadolinium-enhancing lesions on MRI, implying that it may reflect acute inflammation and BBB leakiness. However, because S100B can be elevated in many neurological conditions (including trauma and stroke), its specificity for MS lesions is limited. In practice, S100B is not widely used as an MS biomarker, but it continues to be studied. It might have a niche role in research settings to understand glial responses or to be part of a broader biomarker index. For example, combining S100B with other markers (like NSE or YKL-40) might increase overall sensitivity to detect any neuroglial injury. At present, the clinical impact of S100B in MS is minor compared to more robust markers like NfL or GFAP.

#### 2.3.4. Chitotriosidase-1 (CHIT1)

Chitotriosidase-1 (CHIT1), a glycosyl hydrolase secreted predominantly by activated microglia, has emerged as a robust biomarker candidate for predicting neurodegeneration in multiple sclerosis (MS). Several high-quality studies have consistently demonstrated the prognostic value of CSF CHIT1 levels. Oldoni et al. reported that elevated CSF CHIT1 at diagnosis significantly predicted greater long-term brain tissue damage and disability accumulation in MS patients, distinguishing those with aggressive disease courses [19]. Supporting these findings, Beliën et al. analyzed a large cohort of 192 MS patients and found that CSF CHIT1 was the strongest predictor of early EDSS progression among myeloid biomarkers and histologically localized to lipid-laden microglia within active demyelinating lesions [20]. Furthermore, Cross et al. demonstrated that higher baseline CHIT1 levels correlated positively with the future development of slowly expanding lesions, a marker of chronic active pathology [21]. Collectively, these studies establish CHIT1 as a powerful biomarker reflecting early microglial activation and predicting disease severity, with a strong potential for clinical translation into personalized MS monitoring strategies.

#### 2.3.5. Soluble TREM2 (sTREM2)

Soluble TREM2 (sTREM2), the cleaved extracellular domain of the TREM2 receptor expressed by microglia, has also been investigated as a marker of neuroinflammation and neurodegeneration in MS. Initial studies conducted by Piccio et al. identified elevated CSF sTREM2 levels in patients with active MS, correlating with markers of CNS inflammation [22]. More recently, Ioannides et al. (2021) confirmed higher CSF sTREM2 concentrations in MS patients compared to healthy controls, with positive associations observed between sTREM2, axonal injury markers (neurofilament light chain and phosphorylated neurofilament heavy chain), and disability scores such as EDSS and MSSS [23]. However, in contrast to CHIT1, sTREM2 did not consistently differentiate MS subtypes or predict long-term progression. Genetic studies have also suggested a modest causal relationship between elevated sTREM2 levels and MS risk [24]. Overall, while sTREM2 reflects ongoing microglial activation and appears mechanistically relevant to MS pathology, its prognostic and diagnostic utility remains less definitive compared to CHIT1, necessitating further large-scale longitudinal validation.

### 2.4. Biomarkers of Myelin Biology/Demyelination

#### Myelin Basic Protein

**Figure 1:**
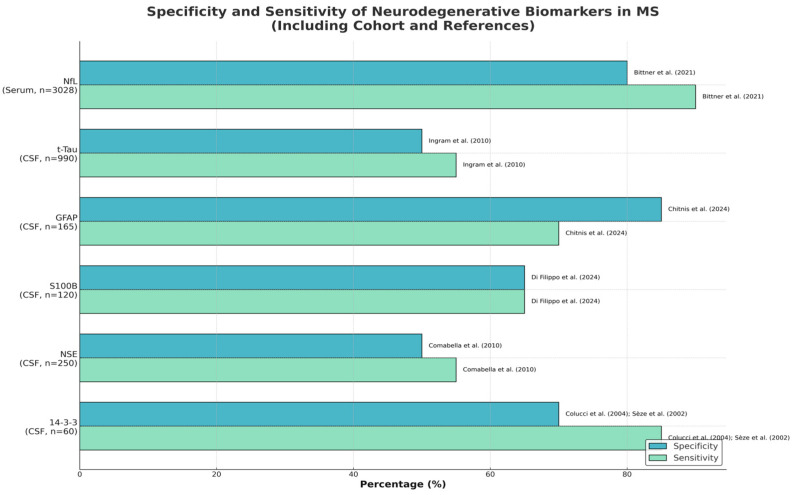
Specificity and sensitivity of neurodegenerative biomarkers in multiple sclerosis, including cohort sizes.

Myelin basic protein (MBP) is produced by the oligodendrocytes from the central nervous system and has been found to be increased in the CSF of patients with MS. MS patients with an acute exacerbation had higher levels than those with slower progressive MS and even higher than those in remission [4]. MBP is a major constituent of the myelin sheath, and, when myelin is destroyed, fragments of MBP can be released into the CSF. Historically, CSF MBP was one of the earliest biochemical markers proposed to track demyelination in MS. During acute MS relapses, especially those with substantial tissue damage, CSF MBP levels can rise transiently. The sensitivity of CSF MBP for MS activity is limited—it tends to be detectable only during acute phases in some patients and often returns to normal in remission. Its specificity for MS is also limited because any demyelinating or CNS destructive process (e.g., traumatic brain injury, other demyelinating diseases) can elevate MBP in CSF [25]. Clinical studies found that adding MBP measurement did not significantly improve MS diagnostic accuracy when oligoclonal bands were already considered [26,27]. As a result, MBP testing in CSF is now rarely used in MS management. Nonetheless, MBP provides evidence of active myelin breakdown, and high MBP levels in CSF correlate with severe attacks or acute disseminated encephalomyelitis. In summary, while MBP is conceptually a direct marker of demyelination, its short-lived elevation and poor disease specificity make it a less useful routine biomarker in MS (Table 1); (Figure 1).

**Neurodegenerative Biomarkers**

Future Biomarkers:

### 2.5. Metabolomic Biomarker

The field of metabolomics, which involves the study of metabolites in biofluids during disease states, is emerging as a powerful approach, as distinct metabolite signatures could be a potential biomarker for disease progression or predictive of the beneficial effect of DMTs in MS. Few studies have outlined a distinct metabolic signature, including serum phospholipids, altered bile acid metabolism, abnormalities in aromatic amino acid metabolism, and pro-resolving lipid mediators in MS compared to healthy subjects, which could be developed as a biomarker for disease and/or novel therapy [4,33]. Some metabolite profiles appear to distinguish progressive MS from relapsing MS or from healthy controls. The sensitivity of individual metabolites is typically low, but, as a panel, metabolomic signatures can achieve useful accuracy. One emerging metabolite of interest is N-acetylaspartate (NAA)—traditionally measured by MR spectroscopy as a marker of neuronal integrity; reduced NAA in CSF could reflect neuronal loss (perhaps indirectly measurable via metabolomics assays). Another example is a pattern of altered tryptophan metabolism, including the kynurenine pathway, which metabolomic analysis can capture [34]. While metabolomics offers a broad view, translating this into a practical biomarker test is challenging. It may be that, in the future, a combination of a few key metabolites will be formulated into a multiplex biomarker panel for MS. As of now, metabolomic biomarkers for MS are in the discovery phase, lacking validation for routine clinical use.

#### 2.5.1. Kynurenines

The kynurenine pathway is involved in neuroinflammation and excitotoxicity. Kynurenic acid, a neuroprotective metabolite, is reduced in MS, whereas quinolinic acid, a neurotoxic metabolite, is increased. This imbalance contributes to axonal damage and mitochondrial dysfunction, making kynurenines promising biomarkers for disease progression and therapeutic targeting [35]. Alterations in this pathway could reflect the balance of neurodegeneration and neuroprotection. Studies indicate that MS patients often have an imbalance in kynurenine metabolites; for instance, increased levels of QUIN (a marker of macrophage/microglia activation) have been observed in MS lesions and CSF [34]. On the other hand, levels of KYNA may be reduced in progressive MS, potentially diminishing neuroprotection [36]. The sensitivity and specificity of kynurenine pathway metabolites as biomarkers are still being evaluated. They are not disease-specific (these metabolites can be altered in other inflammatory neurological disorders), but a distinctive pattern such as a high QUIN:KYNA ratio might be indicative of active MS pathology [34]. Overall, kynurenines are promising research biomarkers that link neuroinflammation to neurodegeneration; they could eventually complement protein biomarkers by providing metabolic insight. However, current data on their diagnostic performance in MS are limited, and assays are not yet standardized for clinical use.

#### 2.5.2. Lipid Biomarkers (Ceramides, Cholesterol, Oxysterols, Phospholipids)

Lipid metabolism plays a crucial role in myelin integrity and neuronal function. Specific sphingolipids and phospholipids have been identified as potential biomarkers for MS progression. Lipidomic profiling can help detect early neurodegenerative changes and assess the impact of therapeutic interventions on lipid metabolism [37]. Lipids are fundamental to myelin and cell membranes, and lipid disturbances have been observed in MS. Demyelination releases cholesterol and other lipids, which are then metabolized into compounds that can be detected in blood or CSF. Certain ceramides and sphingolipids have been found at different levels in MS patients versus controls, implicating these molecules as potential biomarkers of neurodegeneration [38]. For instance, elevated levels of specific ceramide species were associated with more severe disability in some MS cohorts [39]. Oxysterols (oxidized cholesterol derivatives) like 24S-hydroxycholesterol are higher when active demyelination occurs, reflecting CNS cholesterol turnover. Phospholipid profiles in CSF have also been correlated with progressive MS. The sensitivity of individual lipid markers varies, but, collectively, a lipid panel might capture pathological processes such as demyelination or remyelination attempts. Neurodegenerative diseases, MS exhibits a unique lipid signature, though not as pronounced as in primary dementias. Lipid biomarkers also tie into other pathways; for example, high levels of certain lysosomal lipids could indicate microglial activation. The specificity of lipid changes to MS is moderate—many lipid alterations are seen in other neurodegenerative disorders—but combining them with MS-specific clinical context enhances relevance. The use of lipid biomarkers is not yet mainstream in MS, but they represent an emerging area. In the future, tracking lipid changes could complement protein biomarkers, giving a fuller picture of tissue breakdown and repair. For now, they remain largely research tools, requiring more evidence to become part of clinical monitoring.

#### 2.5.3. Extracellular Vesicles (EVs)

EVs can be broken down into microvesicles and exosomes based on size. Microvesicles typically range from 100 to 1000 nanometers (nm), while exosome sizes generally fall within the range of 50 to 150 nm. Exosomes have the capacity to facilitate communication between cells and travel large distances in the body. Thus, they can be used as biomarkers for monitoring MS disease progression and activity as well as therapeutic treatment. Exosomes can be released from T cells to regulate antigen-presenting cells via miRNAs contained within the exosome and act as proinflammatory regulators in rheumatoid arthritis, Grave’s disease, and in MS. EVs are found to be increased in the CSF and plasma of MS patients and have different molecular compositions compared to EVs from healthy individuals [4]. One study found that levels of EVs expressing astrocytic or neuronal markers were higher during MS relapses and correlated with new MRI lesions, suggesting that EV counts could reflect active disease [40]. The sensitivity of EV-based biomarkers can be high when using advanced detection methods, because even subtle ongoing damage might continuously release vesicles. Specificity depends on identifying cell-specific cargo, for example, EVs carrying GFAP or neurofilaments would be more specific to CNS damage. Early results are promising; elevated EVs originating from endothelial cells have been noted in MS and linked to blood–brain barrier breakdown, whereas EVs containing synaptic proteins might indicate neurodegeneration [41]. However, EV analysis is technically complex and not yet standardized. The clinical utility remains exploratory—EV measurements could one day become a liquid biopsy of the CNS, offering a composite view of various cell injuries in MS. Currently, they provide research insights and may improve our understanding of MS pathology and intercellular communication, rather than serving as routine clinical biomarkers.

The following table reunites both actual and future possible biomarkers and their current and possible clinical importance development in MS patients (Table 2).

**CSF vs. Blood-Based Biomarkers: Comparison and Clinical Applications**

**Cerebrospinal fluid (CSF)** has traditionally been the fluid of choice for CNS biomarkers because of its proximity to the brain and spinal cord. CSF levels of neurodegenerative markers are often higher and more directly reflect CNS pathology, making them very sensitive in research settings [42]. For example, before ultrasensitive assays existed, NfL and other proteins were reliably measurable only in CSF. Consequently, an analysis of CSF can yield highly precise insights into neuronal damage [42]. However, lumbar puncture to obtain CSF is invasive and not practical for frequent monitoring. The discomfort experienced by patients, in addition to the inherent risks associated with the procedure, mean CSF biomarkers are typically checked only at diagnosis or in research, rather than serially in routine care [42].

**Blood-based biomarkers** overcome this limitation. These biomarkers have the advantages of being low risk and inexpensive, allowing repeat measurements to track disease course [42]. The main advance enabling blood-based neurodegeneration markers was the development of highly sensitive immunoassays, which has opened new opportunities in MS biomarker utilization—making markers once limited to CSF quantified in blood and accessible for clinical monitoring (e.g., SIMOA) [5]. For instance, serum NfL closely mirrors CSF NfL and has become the first blood biomarker to show solid utility in MS [43]. Blood tests for NfL are being pilot-tested in clinics to inform treatment decisions, and professional guidelines are beginning to consider how to incorporate sNfL into practice (e.g., proposing cutoff values by age) as a tool to monitor MS evolution. The advantages of blood biomarkers are clear: they enable the high-frequency tracking of disease activity/progression, potentially even at every clinical visit, and can be more cost-effective for long-term follow-up [42]. This is particularly useful for assessing treatment response or catching early signs of progression without requiring an MRI each time [30]. Additionally, blood biomarkers can be included in large trials or longitudinal studies with ease, facilitating research on neuroprotective strategies.

**Limitations:** Despite their convenience, blood-based markers have some disadvantages. Concentrations in blood are much lower than in CSF, so results can be influenced by assay precision and variability. There is also the issue of biological noise—blood values might be affected by peripheral factors and are not entirely specific to MS; any significant neuronal damage (trauma, stroke, other neurodegeneration) can raise NfL, and it must be interpreted within context [5]. CSF, by contrast, is a more specific matrix for CNS changes (fewer confounding peripheral sources), but, again, routine CSF draws are impractical. Thus, each has its role: CSF biomarkers excel in diagnostic contexts or one-time assessments (such as confirming MS and gathering a baseline profile of CNS injury, including both neurodegenerative and inflammatory indices), whereas serum biomarkers are ideal for ongoing monitoring and have clear potential in the day-to-day clinical management of MS. Currently, the most common CSF test for MS diagnosis is the IgG oligoclonal band assay, highlighting that neurodegeneration markers in CSF are not yet standard of care [30]. It is worth noting that no biomarker alone dictates management; rather, these tests add a layer of evidence. For instance, elevated serum NfL levels may prompt the consideration of more rapid MRI scans or a re-evaluation of therapeutic interventions. As research progresses and standard reference ranges are defined, we can expect serum-based neurodegenerative biomarkers (like NfL, GFAP) to become part of routine MS care for tracking disease progression and treatment effectiveness, while CSF-based measures are more efficient for certain diagnostic or research applications where maximum specificity is required [5].

## 3. Expanding Clinical Applications of Biomarkers in MS

Biomarkers play a pivotal role in clinical decision making, allowing for enhanced disease monitoring, treatment response assessment, and personalized medicine approaches. Their integration into routine practice could revolutionize MS management.

### 3.1. Diagnosis and Early Detection

Neurodegenerative biomarkers, particularly neurofilament light chain (NfL), have demonstrated high sensitivity in detecting axonal injury at early disease stages [6,13]. Elevated serum NfL levels can differentiate MS from other neurological disorders, providing a non-invasive diagnostic tool [28]. When combined with oligoclonal bands (OCBs) in cerebrospinal fluid, these markers improve diagnostic accuracy [11].

Furthermore, advanced multi-omics approaches integrating transcriptomics, proteomics, and metabolomics are emerging as powerful diagnostic tools. Studies suggest that combining NfL with genetic and metabolic markers could improve early detection accuracy, particularly in individuals at risk of developing MS.

### 3.2. Disease Progression Monitoring

Regular monitoring of NfL levels in serum or cerebrospinal fluid provides real-time insights into ongoing neurodegeneration [44]. Increased levels correlate with brain atrophy and worsening disability, making it a valuable biomarker. Additionally, lipidomic profiling can help identify metabolic changes associated with worsening symptoms [37].

Continuous biomarker assessment, including neuroinflammatory cytokines and glial activation markers, could aid disease tracking. Longitudinal studies demonstrate that dynamic changes in these biomarkers can predict relapses and progressive disease transition, enabling early therapeutic interventions.

### 3.3. Prognostication and Risk Stratification

Kynurenine pathway metabolites, including quinolinic acid, serve as prognostic biomarkers by identifying patients at higher risk for aggressive disease progression [35]. Elevated levels of GFAP and CHI3L1 have also been linked to poorer long-term outcomes [12]. These biomarkers help in risk stratification and guide treatment intensity decisions, ensuring that patients receive the most appropriate and effective treatment for their condition.

Emerging research has explored the potential of gut microbiota-derived metabolites as indicators of MS progression. Alterations in gut microbial composition and associated metabolite levels have shown promise in distinguishing between relapsing and progressive disease phenotypes.

### 3.4. Treatment Response Evaluation

Serum NfL has emerged as a reliable biomarker for assessing treatment efficacy [28]. Patients on high-efficacy disease-modifying therapies (DMTs) such as natalizumab and ocrelizumab exhibit significantly lower NfL levels over time, indicating reduced neuroaxonal damage [13]. Monitoring lipid biomarkers can also provide insights into the metabolic impact of DMTs [37].

Artificial intelligence (AI)-driven biomarker analysis is an evolving field that enables personalized therapeutic adjustments. AI models trained on biomarker datasets can predict treatment responsiveness, reducing trial-and-error in therapy selection.

### 3.5. Personalized Treatment Strategies

The integration of multiple biomarkers allows for a precision medicine approach in MS management [11]. By combining CSF, blood-based, and molecular biomarkers with machine learning algorithms, clinicians can predict individual responses to specific therapies, minimizing adverse effects and optimizing outcomes [37].

**Figure 2:**
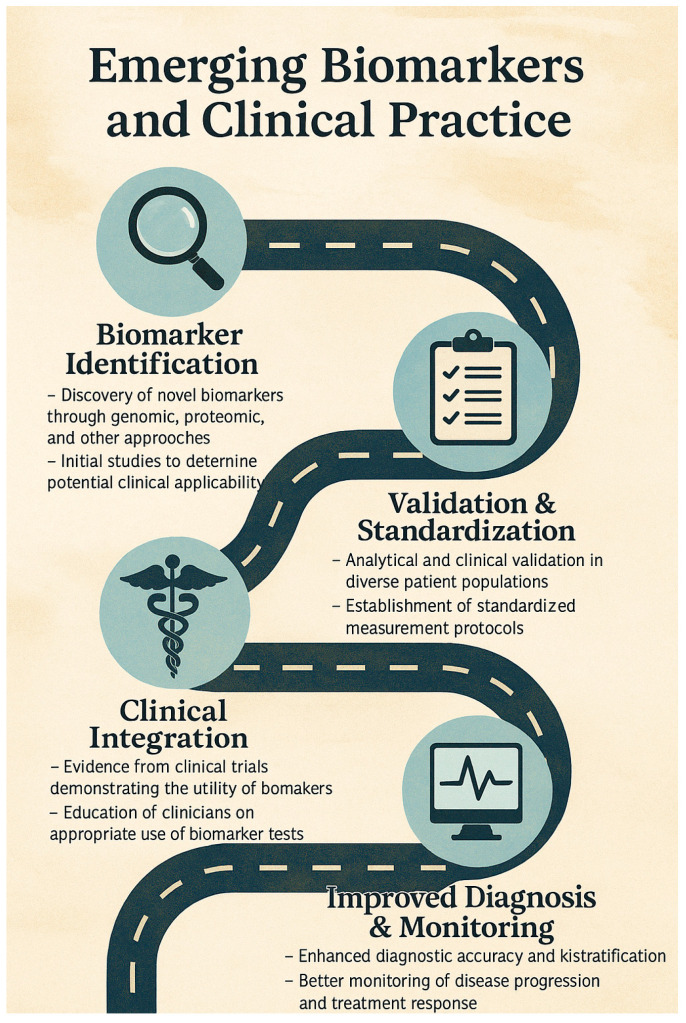
Emerging biomarkers and clinical practice.

Precision medicine approaches now extend to biomarker-guided drug repurposing exploring whether existing medications for neurodegenerative diseases can be repurposed for MS based on shared biomarker profiles (Figure 2).

### 3.6. Expanding Biomarker Research in MS

The future of MS biomarker research focuses on developing multi-biomarker panels that integrate the following:

- Neuroaxonal Injury Markers: NfL, tau protein, and neurogranin.
- Glial Activation Markers: GFAP, CHI3L1, and S100B.
- Neuroinflammatory Markers: Cytokine panels, complement proteins.

Metabolomic Markers: Kynurenines, lipidomic profiles, gut microbiome metabolites.

## 4. Discussion

This review highlights the growing utility of neurodegenerative biomarkers in improving diagnostic precision, tracking disease progression, and optimizing therapeutic strategies in multiple sclerosis (MS). The evidence strongly supports the clinical value of serum and CSF neurofilament light chain (NfL), particularly for monitoring axonal damage and treatment response. Emerging biomarkers, such as glial fibrillary acidic protein (GFAP), chitinase-3-like protein 1 (CHI3L1), and metabolomic indicators, demonstrate promise in refining our understanding of progressive MS phenotypes and tailoring patient-specific care.

However, several challenges remain. Many emerging biomarkers still lack standardized reference ranges, robust longitudinal data, and cross-cohort validation. Interindividual variability, non-specificity to MS, and assay-dependent differences may also confound results. Despite these challenges, integrating multi-biomarker panels—encompassing neuroaxonal, glial, and metabolic indicators—offers a future framework for precision neurology in MS care. The use of biomarkers should complement, not replace, clinical judgment and imaging tools. Further research is necessary to validate these biomarkers across diverse populations and establish consensus guidelines for their routine use.

## 5. Limitations of This Review

This review is narrative in nature and does not include a formal meta-analysis, which may limit the statistical rigor of conclusions. Although comprehensive, the study selection process was not blinded, and publication bias may have influenced the inclusion of more well-known or favorable biomarker studies. Additionally, the heterogeneous nature of included studies—with differences in patient populations, assay techniques, and biomarker thresholds—limits direct comparisons. Finally, rapid advancements in biomarker technologies and bioinformatics mean that the field is evolving, and newer markers may soon alter current clinical paradigms.

## 6. Conclusions

Neurodegeneration significantly contributes to MS-related disability, underscoring the need for reliable biomarkers to monitor disease progression. CSF and blood-based biomarkers, in conjunction with advanced imaging modalities, offer promising paths for early diagnosis, prognostic evaluation, and treatment response evaluation. The analysis of biomarkers such as NfL, GFAP, CHI3L1, tau protein, kynurenines, and lipid markers provide valuable insights into disease evolution. Future research should focus on integrating these biomarkers into clinical practice, developing multi-biomarker panels. By advancing neurodegenerative biomarker research, clinicians will be better equipped to personalize treatment strategies and improve long-term disease management in MS.

## Figures and Tables

**Table 1:** Sensitivity and specificity of current neurodegenerative biomarkers.

Biomarker	Specificity	Sensitivity	Cohort	Reference	Evidence Strength
Neurofilament Light Chain (NfL)	Moderate to High	High	MS patients (serum, n = 3028); multiple studies including Bittner et al. (n = 683)	Bittner et al. (2021) [28] Kapoor et al. (2020) [29] Fox et al. (2021) [30]	High
Total Tau (t-Tau)	Low to Moderate	Variable	MS patients (CSF, n = 990) vs. controls (n = 615)	Ingram et al. (2010) [9]	Moderate
Glial Fibrillary Acidic Protein (GFAP)	High	Moderate	RRMS and PMS patients (CSF, n = 165); Chitnis et al. cohort	Chitnis et al. (2024) [17]	High
S100B	Moderate (Exploratory)	Moderate (Exploratory)	MS patients (CSF, n = 120); single-center cohort	Di Filippo et al. (2024) [7]	Moderate
Neuron-Specific Enolase (NSE)	Low to Moderate	Low to Moderate	RRMS, SPMS, PPMS patients (CSF, n = 250); pooled cohort analysis	Comabella et al. (2010) [14]	Moderate
14-3-3 Protein	High Sensitivity in CIS; Limited Specificity in MS	High in CIS	CIS patients (n = 21); RRMS/SPMS (n = 39); Colucci et al. single-center study	Colucci et al. (2004); [31] Sèze et al. (2002) [32]	Moderate

**Table 2:** Current and future possible neurodegenerative biomarkers regarding clinical utility.

Biomarker	Biological Sample	Significance in MS	Clinical Utility	Clinical Status	References
**CURRENT BIOMARKERS**					
**Neurofilament Light Chain (NfL)**	CSF, Blood	Biomarker of axonal damage; correlates with disease activity and progression.	Diagnostic and Progression Monitoring	Approved	Khalil et al. 2024; [6] Bittner et al. 2021 [28]
**Tau Protein (t-tau, p-tau)**	CSF	Implicated in neurodegeneration and cognitive dysfunction in MS.	Progression Monitoring	Under Validation	Londoño et al. 2022 [10]
**14-3-3 Protein**	CSF	Associated with severe disability and faster MS progression; limited sensitivity.	Progression Monitoring	Under Validation	Colucci et al. 2004 [31]
**Neuron-Specific Enolase (NSE)**	CSF, Blood	Elevated levels during progression phases; neuronal damage marker.	Progression Monitoring	Under Validation	Koch et al. 2015 [16]
**Kynurenines (Kynurenic Acid, Quinolinic Acid)**	CSF, Blood	Imbalance affects neuroinflammation and neurotoxicity.	Progression Monitoring	Exploratory	Sandi et al. 2021; [34] Pukoli et al. 2021 [36]
**Amyloid Precursor Protein (APP)**	CSF, Brain Tissue	Marker of acute axonal injury during demyelination.	Diagnostic and Progression Monitoring	Under Validation	Gehrmann et al., 1995 [15]
**Tubulin Beta (TUB β)**	CSF, Brain Tissue	Associated with neuron regeneration; elevation seen in MS.	Progression Monitoring	Exploratory	Comabella et al. 2010 [14]
**Myelin Basic Protein (MBP)**	CSF	Reflects acute demyelination; increased during relapses.	Diagnostic and Progression Monitoring	Under Validation	Ziemssen et al. 2019 [25]
**Chitinase-3-like Protein 1 (CHI3L1/YKL-40)**	CSF	Associated with glial activation and aggressive MS forms.	Progression Monitoring	Under Validation	Momtazmanesh et al. 2021 [12]; Comabella et al., 2010 [14]
**Glial Fibrillary Acidic Protein (GFAP)**	CSF, Blood	Marker of astrocytic activation; correlates with progressive MS.	Progression Monitoring	Under Validation	Chitnis et al. 2024 [17]; Momtazmanesh et al. 2021 [12]
**S100B Protein**	CSF, Blood	Marker of blood–brain barrier dysfunction; modest diagnostic utility.	Progression Monitoring	Under Validation	Di Filippo et al., 2024 [7]
**Chitotriosidase-1 (CHIT1)**	CSF	Marker of microglial activation; strong predictor of disease severity and brain atrophy.	Prognostic Monitoring	Under Validation	Oldoni et al. 2020 [19]; Beliën et al. 2024 [20]
**Soluble TREM2 (sTREM2)**	CSF	Reflects microglial activation and axonal injury; prognostic associations suggested.	Progression Monitoring	Exploratory	Ioannides et al. 2021 [23]; Piccio et al. 2008 [22]
FUTURE BIOMARKERS					
**Extracellular Vesicles (EVs)**	CSF, Blood	Altered in MS; potential for monitoring disease activity.	Diagnostic and Progression Monitoring	Exploratory	Manna et al. 2024 [40]; Gutiérrez-Fernández et al. 2021 [41]
**Metabolomics (General Metabolite Panels)**	Blood, CSF	Emerging metabolic markers linked to progression and therapy response.	Diagnostic and Progression Monitoring	Exploratory	Singh et al. 2019 [33]
**Kynurenines (Detailed Pathway)**	CSF, Blood	Disruption in kynurenine pathway contributes to neurodegeneration.	Progression Monitoring	Exploratory	Mey et al. 2023 [35]; Sandi et al. 2021 [34]
**Lipid Biomarkers (Ceramides, Cholesterol, Oxysterols, Phospholipids)**	Blood, CSF	Changes in lipid composition associated with myelin integrity and neurodegeneration.	Diagnostic and Progression Monitoring	Exploratory	Wei et al. 2024 [38]; Jiao et al. 2024 [37]

## Data Availability

The original contributions presented in this study are included in the article. Further inquiries can be directed to the corresponding author(s).
